# COVID-19 and Semen Fluid Parameters, a Retrospective Study from Infertility Clinics

**DOI:** 10.3390/life12122076

**Published:** 2022-12-10

**Authors:** Zina M. Al-Alami, Soha Albeitawi, Maha S. ALNatsheh, Khaled Albakri, Hussein Qublan, Nadia Muhaidat, Mariam Ahmad Abuhalaweh, Maen Monketh AlRawashdeh, Hiba Alqam

**Affiliations:** 1 Medical Laboratory Sciences Department, Faculty of Allied Medical Sciences, Al-Ahliyya Amman University, Amman 19328, Jordan; 2 Clinical Science Department, Faculty of Medicine, Yarmouk University, Irbid 21163, Jordan; 3 IVF Department, Al-Kindi Hospital, P.O. Box 851575, Amman 11185, Jordan; 4 Faculty of Medicine, The Hashemite University, Zarqa 13133, Jordan; 5 Irbid Specialty Hospital IVF Center, Irbid Specialty Hospital, Irbid 21110, Jordan; 6 Department of Obstetrics and Gynecology, School of Medicine, The University of Jordan, Amman 11942, Jordan; 7 Obstetrics and Gynecology Department, Al-Kindi Hospital, P.O. Box 851575, Amman 11185, Jordan; 8 School of Medicine, RCSI University of Medicine and Health Sciences, D02 YN77 Dublin, Ireland; 9 Obstetrics and Gynecology Department, Islamic Hospital, Al-Abdali, King Hussein street, Amman 11942, Jordan

**Keywords:** COVID-19, fertility, infection, Jordan, SARS-CoV-2, SFA, sperm, vaccines

## Abstract

The study of the effects of SARS-CoV-2 infection and/or vaccination on semen fluid analysis (SFA) parameters is still incomplete. The aim of this study is to assess the effect of COVID-19 infection and vaccination on sperm parameters for a sample of individuals visiting multi-infertility clinics in Jordan. SFA records were collected retrospectively between September and November 2021 and analyzed using Jamovi software (version 2.2.5 for Windows); *p*-values < 0.05 were considered statistically significant. Sperm concentration, progressive motility, normal morphology, and semen liquefaction time, volume, and viscosity were compared among two data categories. In the first category of data, SFA records from 354 participants were separated into four groups: only vaccinated, infected and vaccinated, neither infected nor vaccinated, and only infected. In the other category, SFA from 49 subjects before their infection and/or vaccination and after were classified into the same mentioned groups and analyzed. There were no statistically significant differences between the studied parameters in the SFA records in the first data category and the second. Nevertheless, the sperm concentration was higher among vaccinated subjects compared to unvaccinated ones (*p* = 0.04). It is concluded that SARS-CoV-2 infection and vaccines have no negative effects on SFA parameters.

## 1. Introduction

Many humans around the world have been infected by the coronavirus SARS-CoV-2, which caused coronavirus disease 2019 (COVID-19) [1]. Coronaviruses usually cause cough, fever, and shortness of breath and might lead to severe symptoms such as pneumonia [2]. The virus continues to adversely affect social and economic situations and, most crucially, human health by affecting a variety of organs [3]. About 80% of SARS-CoV-2 infections are considered mild, while 5% could be critical and 3% could lead to mortality [4]. 

Angiotensin-converting enzyme 2 (ACE2)—the receptor that has a role in SARS-CoV-2 invasion—is highly expressed in the genitourinary tract and the testes [4,5]. Studies have reported that SARS-CoV-2 is not present in the semen of individuals recovering from COVID-19 [6] or patients with a mild form of COVID-19 in their acute stage of the disease [7]; furthermore, some other results implied that the detection of viral RNA of SARS-CoV-2 is actually rare in semen, yet its RNA could be found in semen even in a small sample size [5,8].

Recent review articles and studies summarized the effects of SARS-CoV-2 infection on semen parameters and showed that the infection caused a significant decrease in some semen parameters, particularly number and motility, and an insignificant decrease in others, and correlated the severity of COVID-19 infection and the recovery time with semen quality [9,10,11,12]. Nevertheless, one of the most recent systematic reviews of screened articles that included 1960 controls and 2106 patients showed a temporary negative effect of SARS-CoV-2 infection on male fertility that gradually increased back to normal as patients recovered [13], and SFA parameters returned to normal values within three months in cases of moderate to severe infections [14]. Nonetheless, a new study of 594 patients implied that sperm quality did not change during the pandemic when evaluated against values before the pandemic [15].

COVID-19 vaccines became one of the most vital tools for COVID-19 prevention. They have been accessible in the fight against the pandemic since December 2020 [16]. Since then, the newly developed vaccines have been accompanied by conspiracy theories and rumors [17]. For safety assessment, and to ensure that the vaccines have no negative impact on health, many adverse effects were reported and analyzed. Consequently, several articles and reviews have studied the impacts of COVID-19 vaccines on male infertility, and they stated that vaccines against SARS-CoV-2 have no pathological effects on male fertility or on spermatogenesis, and seminal fluid analysis parameters did not differ among vaccinated and nonvaccinated groups [18,19,20,21].

The continuing anxiety about health posed by COVID-19 and the impact of SARS-CoV-2 infection and vaccination on male reproductive health needs to be thoroughly investigated; any new report related to this issue will be considered a necessary addition to the literature. However, until now, despite the published reports studying the impacts of COVID-19 infection and/or vaccination on male reproduction and on semen parameters, the results are still described as divisive and debated. Scientific evidence of their short-term and/or long-term effects are so far considered hot scientific topics. Thus, this work was conducted to assess the effect of COVID-19 infection and vaccination on a sample of Jordanian individuals visiting multi-infertility clinics in Jordan. In this retrospective study, data about pre- and postinfection and data about pre- and postvaccination were reported and analyzed against semen fluid parameters.

## 2. Materials and Methods

### 2.1. Data Collection

Semen fluid analysis (SFA) records from four infertility units in Jordan were retrospectively reviewed. Data were collected between September and November 2021 from a convenient sample of individuals who visited the units, and samples were used irrespective of the reason for their attendance. 

The data were classified into two categories. In the first category of data, SFA data from 354 participants were classified into four groups: individuals who were only vaccinated, individuals who were vaccinated and infected with the virus, individuals who were neither vaccinated nor infected, and individuals who were only infected with the virus. 

In the other category, data were collected from 49 subjects who visited one infertility unit before their infection and/or vaccination and after. The groups were classified as individuals neither infected nor vaccinated, vaccinated only, infected only, and both infected and vaccinated—the same as the first category.

The collected SFA data included sperm concentration, sperm progressive motility, semen liquefaction time, ejaculate volume, normal forms existing within the semen, and ejaculate viscosity.

### 2.2. Types of Vaccines Offered in Jordan

The COVID-19 vaccination campaign in Jordan started in January 2021. The available vaccines were Pfizer (Pfizer-BioNTech (BioNTech, Mainz, Germany), AstraZeneca–Oxford (Oxford University and British-Swedish company AstraZenec, UK), Sinopharm (Sinofarm, Beijing, China) (BBIBP-CorV COVILO), and Sputnik V (The Gamaleya National Center of Epidemiology and Microbiology, Russia), also known as Gam-COVID-Vac. 

### 2.3. Statistical Analysis

Data were analyzed using Jamovi software (Sydney, Australia) (version 2.2.5 for windows). The variables were described using median (interquartile range (IQR)) and number (%) for continuous and categorical data, respectively. To assess the difference between two or more categorical data points, the chi-square test was used. Further, Wilcoxon and Kruskal–Wallis tests for abnormally distributed continuous variables were used. The results were considered significant when the *p*-value ˂ 0.05. 

### 2.4. Ethical Approval

All research was performed in accordance with relevant guidelines and regulations and in accordance with the Declaration of Helsinki. Patient consent was waived because the study is a retrospective review of medical records. Institutional Review Board (IRB) approval was obtained from the Ethical Reviewing Board at the Faculty of Pharmacy and the Faculty of Allied Medical Sciences at Al-Ahliyya Amman University (approval number: AAU/11/5/2020-2021). 

## 3. Results

In the first category of data, a total of 354 participants were included, with the mean age of the participants being 35.6 ± 7.96. The majority of them were vaccinated only (61%), while the others were infected and vaccinated (23.4%), infected only (8.5%), and neither infected nor vaccinated (7.1%). The types of vaccines taken by the participants were Pfizer (44.1%), Sinopharm (31.4%), AstraZeneca (7.9%), and Moderna (0.3%), while none of them received Sputnik V.

According to Table 1, there was no significant difference between the four groups in terms of total sperm count, concentration, progressive motility, and volume (*p*-value ≥ 0.05), while there was a significant difference in terms of normal forms existing within the semen (*p*-value = 0.04). The chi-square test showed that viscosity and liquefaction were not significantly different between the four groups (*p*-value = 0.74, *p*-value = 0.95, respectively) (Table 1).

In addition, there were no statistically significant differences between the infected individuals (including vaccinated and unvaccinated) and the uninfected individuals (including vaccinated and unvaccinated) in terms of total sperm count, concentration, progressive motility, liquefaction, volume, normal forms existing within the semen, and viscosity (*p*-value ≥ 0.05). Additionally, unvaccinated patients had a higher concentration than vaccinated ones (*p*-value = 0.04). However, there was no significant difference regarding other outcomes (*p*-value ≥ 0.05) (Table 1).

Regarding the types of vaccine given to the participants, there were no significant differences between the different types of vaccines in total sperm count, sperm progressive motility, normal forms existing within the semen, sample concentration, or liquefaction time, volume, or viscosity (*p*-value ≥ 0.05; Table 2).

In the second category of data, 49 participants, whose data were collected before and after infection and before and after vaccination, were included in the study with a mean age of 40.48 ± 8.79 (Table 3).

There were no significant differences between the pre- and post values of SFA in sample volume, liquefaction time, or concentration, or in the percentages of immotile sperms, sperm progressive motility, and normal forms. In addition, there were no statistically significant differences between infected and uninfected or vaccinated and unvaccinated participants regarding the previously mentioned parameters (Table 4).

## 4. Discussion

For the Jordanian public, one of the most important concerns about COVID-19 infection and vaccination is their potential impact on human health, including fertility and reproduction. Global publications about this issue have been many. Some studies were about the impact of COVID-19 infection itself on male fertility, while others explored the SARS-CoV-2 vaccination effects on semen parameters and comparison of data from individuals before and after infection and/or vaccination. 

In the current study, the semen parameters within two categories of data were described and compared: firstly, in a sample of 354 individuals who were either vaccinated only, infected and vaccinated, neither infected nor vaccinated, or infected only; and secondly, in a sample of 49 participants with SFA records before and after infection and/or before and after vaccination. The results in this study revealed that the COVID-19 infection or vaccination did not affect the sperm parameters of the World Health Organization (WHO) characteristics between infected and noninfected individuals or vaccinated and nonvaccinated individuals or between the same subjects before they were infected or vaccinated and after.

Previous publications showed scandalous results; the results of the current study did not corroborate many of them. For example, on the one hand, Aksak et al. [22] reported that the sperm concentration of men who were never infected was significantly higher, giving an explanation that the fever might be the cause behind that [23]. On the other hand, scientists started correlating the change in sperm parameters with the stage of symptoms and relating them to short-term and long-term change. Another report was by Holtmann et al. [24], who reported differences in sperm parameters in moderate COVID-19 cases. Later, in a case study published in February 2021, it was obvious that a mild COVID-19 infection resulted in long-term alterations in sperm morphology and sperm DNA integrity and might take more than four months to return to the basal state of sperm parameters [25]. Then again, Guo et al. [26] reported direct evidence of reversible and temporary reductions in sperm concentration, counts, motility, and progressive motility in patients who had recovered from COVID-19 after admission to hospital. Additionally, total motile sperm count decreased significantly after a mild infection compared to the preinfection readings [27]. Deterioration of semen quality and fertility potential was shown in the short-term after SARS-CoV-2 infection [12]. Moderate COVID-19 infection that require hospitalization could temporarily affect sperm motility [28]. 

However, what was shown in the current study was also concluded by most of the previous publications that said that the infection showed no negative effects on sperm parameters. For instance, in a study in March 2020, the semen parameters of eight out of twelve patients had normal semen characteristics after COVID-19, and the total mobile sperm count in two out of three patients showed a slight decrease after COVID-19 infection when compared to their previous records [29]. Moreover, in early 2021 in Turkey, SFA of two groups were compared. In this study, 100 men who had been infected and recovered and 100 men who never had COVID-19 were studied. There were no statistically significant differences between the two groups in terms of sperm motility and morphology [22]. In addition, Pazir et al. [27] reported that there were no significant differences in semen parameters before and after COVID-19 in terms of semen volume, sperm concentration, and progressive motility. Likewise, in a recent study by Vahidi et al. [30], the semen analyses between patients in the acute and clinical recovery stages showed no significant differences between the two groups in semen count or motility parameters. Additionally, in their retrospective study, Wang et al. [31] studied the semen parameters for male partners in couples undergoing in vitro fertilization cycles in a hospital in Wuhan, China, between May 2020 and February 2021. They provided that there were no significant differences in terms of concentration and before and after infection.

The Jordanian National Vaccination Campaign to fight COVID-19 started in January 2021 and was carried out by the Ministry of Health and the National Centre for Security and Crises Management. Although vaccination is not mandatory, every individual living in Jordan is eligible to receive the COVID-19 vaccine, which is offered for free. As mentioned previously, the vaccines that are available in Jordan are Pfizer (BioNTech), Oxford-AstraZeneca (ChAdOx1-S recombinant), Sinopharm (BBIBP-CorV COVILO), AstraZeneca, and Sputnik V, also known as Gam-COVID-Vac.

In this study, an experiment to identify potential detrimental effects of different types of vaccines on male fertility was designed. As was shown in the results section, vaccines had no effect on semen parameters. The values stated in the current study and the previous studies suggest that vaccinations are safe, with no obvious harmful effects on sperm parameters. To date, there are several studies that were conducted to study the relationship between seminal parameters and the two mRNA vaccines, BNT162b2 (Pfizer-BioNTech) and mRNA-1273 (Moderna). The research groups found no significant abnormalities in any spermatozoa parameter [32,33]. In addition, sperm parameters were similar between men who completed two doses of inactivated COVID-19 vaccines, such as Sinovac and Sinopharm, and those who were not vaccinated [34,35]. Moreover, Reschini et al. [36] also stated that COVID-19 vaccinations with mRNA or viral vector vaccines did not affect sperm quality, and Ali et al. [37] revealed that vaccinations have no effect on seminal parameters. 

Most importantly, Zhu et al. [35] mentioned that there were small fluctuations in some semen parameters, namely, volume, sperm concentration, progressive motility, and total progressive motile count, after the first dose of an inactivated COVID-19 vaccine, but the parameters return to the prevaccination level after the second dose. In their study, the sperm concentration (mean ± SD) increased from 55.6 million sperms to 61.9 million sperms after the first injection and decreased to 55.9 million sperms after the second injection, which is consistent with the current study (Table 1).

Regarding the second category of data, the data were also consistent with the previously published research. The values specified in the current study and the previous publications provide that the infection and vaccination did not change the semen parameters for individuals and had no noticeable harmful effects. In fact, semen parameters did not change before and after infection [36], and there were no differences between the patients who visited the urology clinic before and during the pandemic in semen volume or rates of normospermia or pathological spermiogram findings [15]. Additionally, Elhabak et al. [38] concluded that COVID-19 vaccines on semen parameters are safe when comparing the semen parameters from individuals before and after receiving the AstraZeneca COVID-19 vaccine or Sinopharm (BBIBP-CorV COVILO).

Including the relatively small sample size and because the current study was a retrospective study, there were some limitations in the current study. Principally, no information about the classification of the severity of the infection was available, so classification of the patients into having acute, moderate, or mild symptoms was not applicable. Besides that, clear data about the dates of having the first dose and the second dose of vaccination were not available. Therefore, data could not be analyzed according to date and, hence, according to long-term effects and short-term effects.

Nevertheless, the current study has several strengths. Particularly, until now, there was an incomplete view of whether semen parameters are affected by SARS-CoV-2 infection or not, since most of the studies are frequently limited by no preinfection or prevaccination control samples. In the current study, data that described the semen characteristics of COVID-19 patients before and after infection were provided and compared between infection and vaccination consequences on male fertility in Jordan; no previous similar reports were published previously from Jordan. Another point is that it adds more evidence that neither COVID-19 infection nor vaccination affect male fertility or sperm parameters. This report is considered one of the few studies that compared semen parameters in infected with noninfected (control) individuals, vaccinated and nonvaccinated (control) individuals, and individuals before and after infection. Additionally, data about the same individuals before the pandemic and what happened to their SFA readings after infection or vaccination, or both, were provided.

Future follow-up studies would be necessary to understand if any changes in semen parameters persist and for how long. Other studies could be about the virus variant and its correlation with male fertility. The major concern in such future studies will be that individuals who have been neither infected nor vaccinated might be rare. Accordingly, research on semen samples before and after and in the long term will be quite impossible to find, although they are vital to understand how COVID-19 might affect sperm parameters. 

## 5. Conclusions

According to the available data, SARS-CoV-2 infection and SARS-CoV-2 vaccines have no negative effects on semen fluid analysis parameters. 

## Figures and Tables

**Table 1:** Semen fluid analysis parameters of individuals classified in different groups.

Group		Volume (mL) Median (Q1, Q3)	Concentration (10^6^/μL) Median (Q1, Q3)	Total Sperm Count * (Sperms in Ejaculate) Median (Q1, Q3)	Progressive Motility (%) Median (Q1, Q3)	Normal Forms (%) Median (Q1, Q3)
	Parameter					
Neither infected nor vaccinated		2.50 (2.00, 3.00)	18 (9, 29)	45 (18, 74)	10 (0, 25)	2.00 (1.0, 3.0)
Infected only		2.50 (2.00, 4.38)	22 (8, 49)	57 (23, 114)	20 (5, 34)	7.00 (2.0, 10.0)
Vaccinated only		3.00 (2.00, 3.50)	28 (12, 55)	75 (28, 149)	20 (5, 35)	2.00 (1.0, 9.5)
Both infected and vaccinated		3.00 (2.50, 3.50)	30 (12, 47)	88 (36, 161)	15 (5, 30)	3.00 (2.0, 7.5)
*p*-value		0.49	0.17	0.05	0.38	0.04
Infected group		3.00 (2.00, 3.50)	27 (12, 50)	68 (30, 144)	20 (5, 35)	2.00 (1.0, 8.0)
Non infected group		3.00 (2.00, 3.50)	27 (10, 48)	72 (27, 145)	15 (5, 30)	3.0 (2.0, 10.0)
*p*-value		0.36	0.79	0.74	0.83	0.12
Vaccinated group		2.50 (2.00, 3.75)	22 (8, 34)	76 (30, 150)	20 (2, 30)	2.50 (1.0, 10.0)
Unvaccinated group		3.00 (2.00, 3.50)	30 (12, 52)	54 (20, 90)	15 (5, 35)	3.00 (1.0, 8.0)
*p*-value		0.30	0.04	0.01	0.34	0.90

* Total sperm count = (sperm concentration × semen volume).

**Table 2:** Semen fluid analysis parameters in individuals after using different types of vaccines.

Parameter	Pfizer	AstraZeneca	Sinopharm	Moderna	*p*-Value
Volume (mL) Median (Q1, Q3)	2.80 (2.00, 3.50)	3.00 (2.00, 4.12)	3.00 (2.45, 4.00)	3.00 (3.00, 3.00)	0.31
Concentration (10^6^/μL) Median (Q1, Q3)	30 (12, 49)	37 (20, 51)	26 (12, 57)	19 (19, 19)	0.76
Total sperm count * (Sperms in ejaculate) Median (Q1, Q3)	69 (27, 144)	104 (58, 148)	80 (31, 174)	57 (57, 57)	0.35
Progressive Motility % Median (Q1, Q3)	15 (5, 35)	22 (3, 35)	15 (5, 30)	30 (30, 30)	0.89
Normal forms (%) Median (Q1, Q3)	3.00 (1.0, 8.0)	2.00 (1.0, 2.8)	3.00 (1.0, 11.0)	1.00 (1.0, 1.0)	0.06

* Total sperm count = (sperm concentration × semen volume).

**Table 3:** The Characteristics of the Participants Whose Data were Collected Before and After Infection or Vaccination.

Number of individuals		N = 49
Age Mean ± standard deviation (SD)		40.48 ± 8.79
		N (%)
Groups	Neither infected nor vaccinated	3 (6.1)
	Vaccinated only	28 (57)
	Infected only	4 (8.2)
	Both infected and vaccinated	14 (29)
COVID-19 infection	No	31 (63)
	Yes	18 (37)
Received COVID-19 vaccine	No	7 (14)
	Yes	42 (86)
Type of vaccine	AstraZeneca	5 (12)
	Moderna	1 (2.4)
	Pfizer	27 (64)
	Sinopharm	6 (14)
	Sputnik	1 (2.4)
	Pfizer	2 (4.8)

**Table 4:** Changes That Occurred in the Semen Parameters After the Individuals Were Infected or Vaccinated.

Variables	Neither Infected Nor Vaccinated (N = 3)	Vaccinated Only (N = 28)	Infected Only (N = 4)	Both Infected and Vaccinated (N = 14)	*p*-Value
Change in sample volume					0.35 ^1^
Mean ± SD	–0.2 ± 0.3	–0.2 ± 1.0	–0.1 ± 0.9	0.3 ±1.0	
Range	–0.5–0.0	–1.5–2.5	–1.0–1.0	–1.0–3.0	
Change in sample liquefaction time					NA ^1^
Mean ± SD	0.0 ± 0.0	0.0 ± 0.0	0.0 ± 0.0	0.0 ± 0.0	
Range	0.0–0.0	0.0–0.0	0.0–0.0	0.0–0.0	
Change in sample concentration					0.21 ^1^
Mean ± SD	–1.7 ± 5.5	–9.0 ± 16.3	3.2 ± 12.6	–1.4 ± 22.6	
Range	–7.0–4.0	–60.0–33.0	–10.0–20.0	–42.0–50.0	
Change in sperms progressive motility					0.69 ^1^
Mean ±SD	–7.7 ± 8.7	–5.8 ± 13.2	–3.8 ± 12.5	–3.1 ± 11.1	
Range	–15.0–2.0	–20.0–30.0	–20.0–10.0	–25.0–10.0	
Change in immotile sperms					0.48 ^1^
Mean ± SD	6.7 ± 7.6	–0.4 ± 18.5	–22.0 ± 45.6	–6.2 ± 27.5	
Range	0.0–15.0	–60.0–30.0	–90.0–7.0	–95.0–17.0	
Change in sperms with normal forms					0.51 ^1^
Mean ±SD	–0.3 ± 0.6	–1.3 ± 1.7	–9.8 ± 18.2	–0.6 ± 1.3	
Range	–1.0–0.0	–8.0–1.0	–37.0–1.0	–2.0–2.0	

Numbers with minus signs signify that the mean value after the infection or vaccination was greater than the mean value before infection or vaccination. SD = standard deviation. NA = not applicable. ^1^ Kruskal–Wallis was used to assess the difference, as the variables were abnormally distributed.
